# Visuo‐manual tracking: does intermittent control with aperiodic sampling explain linear power and non‐linear remnant without sensorimotor noise?

**DOI:** 10.1113/JP274288

**Published:** 2017-10-01

**Authors:** Henrik Gollee, Peter J. Gawthrop, Martin Lakie, Ian D. Loram

**Affiliations:** ^1^ School of Engineering University of Glasgow Glasgow UK; ^2^ Systems Biology Laboratory, Melbourne School of Engineering University of Melbourne Melbourne Australia; ^3^ School of Sport and Exercise Sciences University of Birmingham Birmingham UK; ^4^ School of Healthcare Science Manchester Metropolitan University Manchester UK

**Keywords:** motor control, intermittent control, variability

## Abstract

**Key points:**

- A human controlling an external system is described most easily and conventionally as linearly and continuously translating sensory input to motor output, with the inevitable output remnant, non‐linearly related to the input, attributed to sensorimotor noise.
- Recent experiments show sustained manual tracking involves repeated refractoriness (insensitivity to sensory information for a certain duration), with the temporary 200–500 ms periods of irresponsiveness to sensory input making the control process intrinsically non‐linear.
- This evidence calls for re‐examination of the extent to which random sensorimotor noise is required to explain the non‐linear remnant.
- This investigation of manual tracking shows how the full motor output (linear component and remnant) can be explained mechanistically by aperiodic sampling triggered by prediction error thresholds.
- Whereas broadband physiological noise is general to all processes, aperiodic sampling is associated with sensorimotor decision making within specific frontal, striatal and parietal networks; we conclude that manual tracking utilises such slow serial decision making pathways up to several times per second.

**Abstract:**

The human operator is described adequately by linear translation of sensory input to motor output. Motor output also always includes a non‐linear *remnant* resulting from random sensorimotor noise from multiple sources, and non‐linear input transformations, for example thresholds or refractory periods. Recent evidence showed that manual tracking incurs substantial, serial, refractoriness (insensitivity to sensory information of 350 and 550 ms for 1st and 2nd order systems respectively). Our two questions are: (i) What are the comparative merits of explaining the non‐linear remnant using noise or non‐linear transformations? (ii) Can non‐linear transformations represent serial motor decision making within the sensorimotor feedback loop intrinsic to tracking? Twelve participants (instructed to act in three prescribed ways) manually controlled two systems (1st and 2nd order) subject to a periodic multi‐sine disturbance. Joystick power was analysed using three models, continuous‐linear‐control (CC), continuous‐linear‐control with calculated noise spectrum (CCN), and intermittent control with aperiodic sampling triggered by prediction error thresholds (IC). Unlike the linear mechanism, the intermittent control mechanism explained the majority of total power (linear and remnant) (77–87% *vs*. 8–48%, IC *vs*. CC). Between conditions, IC used thresholds and distributions of open loop intervals consistent with, respectively, instructions and previous measured, model independent values; whereas CCN required changes in noise spectrum deviating from broadband, signal dependent noise. We conclude that manual tracking uses open loop predictive control with aperiodic sampling. Because aperiodic sampling is inherent to serial decision making within previously identified, specific frontal, striatal and parietal networks we suggest that these structures are intimately involved in visuo‐manual tracking.

## Key points

- A human controlling an external system is described most easily and conventionally as linearly and continuously translating sensory input to motor output, with the inevitable output remnant, non‐linearly related to the input, attributed to sensorimotor noise.
- Recent experiments show sustained manual tracking involves repeated refractoriness (insensitivity to sensory information for a certain duration), with the temporary 200–500 ms periods of irresponsiveness to sensory input making the control process intrinsically non‐linear.
- This evidence calls for re‐examination of the extent to which random sensorimotor noise is required to explain the non‐linear remnant.
- This investigation of manual tracking shows how the full motor output (linear component and remnant) can be explained mechanistically by aperiodic sampling triggered by prediction error thresholds.
- Whereas broadband physiological noise is general to all processes, aperiodic sampling is associated with sensorimotor decision making within specific frontal, striatal and parietal networks; we conclude that manual tracking utilises such slow serial decision making pathways up to several times per second.

**Abbreviations**
CC: continuous‐linear‐control
CCN: continuous‐linear‐control with calculated noise spectrum
IC: intermittent control
LQR: linear‐quadratic regulator
GMM: Gaussian mixture model
PSD: power spectral density
SPM‐1D: 1‐d statistical parametric mapping

## Introduction

Visually guided manual tracking in humans and primates has been studied extensively. This task engages processes common to many activities of daily living including visually guided manipulation of tools such as precision surgery and playing musical instruments, general reaching and pointing, and control of vehicles and computer games.

**Figure 1:**
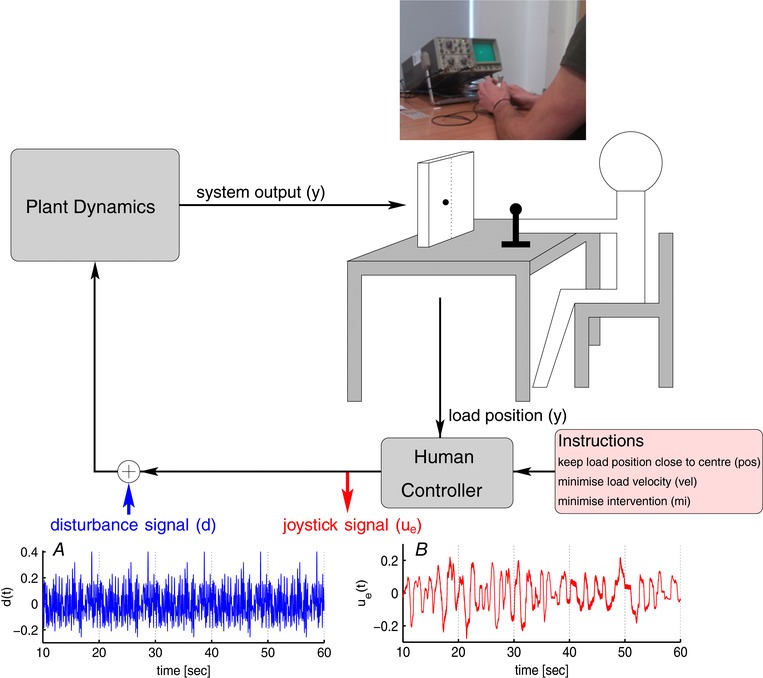
Experimental setup Participants sat at a table, manipulating a table‐supported, sensitive, uni‐axis joystick with their self‐chosen hand using continuous contact. Position of the system output, moving left–right within a horizontal line, was displayed 50 cm away on an oscilloscope (CRO) of full scale range, 10 cm. The person controls the system output position displayed on a screen using a joystick in accordance with different instructions. The virtual systems included an unstable (2nd order) system with an unstable time constant of 0.9 s and no friction or damping and a stable, first order system. An external periodic multi‐sine disturbance signal, *d* (panel *A*, extract of 5 periods of 10 s each shown) is added to the time‐varying joystick signal, $u_{e}$ (panel *B*). [Color figure can be viewed at wileyonlinelibrary.com]

The engineering control basis of manual tracking was established in the 1960s. In typical experiments, the human operator controls a dynamic system. The dynamic system is subject to a disturbance which allows analysis of the manual control signal and identification of a control model representing the ‘human in the loop’ operator. The manual control signal is typically the continuous signal arising from a joystick (Fig. 1). For identification of the human controller, the disturbance is regarded as a known input. The joystick power spectrum includes a component which is related linearly to that input and a non‐linear remnant component. The linear component can be reconstructed from that input by scaling and shifting. Potentially, the non‐linear remnant includes stochastic parts, i.e. those which are random, and deterministic parts, i.e. those reconstructable from that input by a non‐linear rule. To be more specific, the non‐linear remnant includes pure *noise* from multiple sources, *non‐linear operations* including aperiodic sampling, thresholds and refractory periods, and *non‐steady behaviour* including changes through time in control processes (Levison *et al*. 1969; Jagacinski & Flach, 2003; George, 2008). Broadband random noise is associated with all biological processes (Newell *et al*. 2006). These noise sources are assumed to arise from basic physiological sensory and motor processes. Apart from being proportional to the signal variance, this physiological noise has been found to be independent of system dynamics, controller parameters, disturbance amplitude and disturbance bandwidth (Levison *et al*. 1969). Aperiodic sampling is a non‐linear operation using thresholds and refractory periods to determine when sampling occurs. Refractory, threshold related motor decision making is associated with specific parietal, prefrontal and striatal pathways (Dux *et al*. 2006; Gold & Shadlen, 2007; Beck *et al*. 2008; Carpenter *et al*. 2009; Frank, 2011).

At one level, our question concerns the relative merits of explaining the non‐linear remnant using noise and using aperiodic sampling. At a deeper level, our question concerns the prevalence and hierarchical level of motor decision making within sensorimotor control. Discrete response selection tasks and continuous sensorimotor activities are sometimes regarded as different processes with distinct neural substrates (Hardwick *et al*. 2013). However, discrete, motor decision making can extend potentially to the selection of goals, of actions to achieve a selected goal, of movements to achieve a selected action and of sub‐movements to perform a selected movement (Haber *et al*. 2000). Determining whether implementation of ongoing sensorimotor control is dependent upon serial motor decision making, occurring within specific local pathways, is relevant to understanding the linked degradation of motor decision making and sensorimotor control in neurological conditions and ageing (Harrison *et al*. 1995; Delbaere *et al*. 2010; Rochester *et al*. 2014). If motor decision making is limited to a higher, ‘executive’ level *outside* the feedback loop, continuous manual tracking may require no decision making and no refractoriness, once the task is initiated. Our question concerns the strength of the evidence for serial motor decision making, at the level of sub‐movements and on the temporal scale of 200–500 ms, lying *within* the sensorimotor feedback loop implementing continuous manual tracking.

In 1969, Kleinman *et al*., studying human manual control of external systems, demonstrated that the human response could be represented by a transport delay and linear continuous optimal controller including a cascade combination of Kalman filter (observer), least mean squared predictor and transfer function representing the lags of the neuromuscular system (Kleinman, 1969; Levison *et al*. 1969; Kleinman *et al*. 1970). Following Kleinman *et al*., many investigators have considered various stochastic and deterministic sources of the non‐linear remnant in human motor control. However, arguably, Kleinman *et al*. provided an explanation of experimental data subsequently unsurpassed by alternative explanations. Their extensive experimentation and analysis showed that the non‐linear remnant could be reproduced by adding an observation vector of linearly independent noise processes (Levison *et al*. 1969). With foveal display viewing, the component noise processes are proportional to the variances of the displayed quantities. The remnant is considered a smooth function of frequency (Levison *et al*. 1969). We acknowledge that many authors have considered additive and state‐dependent noise, the interaction of noise with complex systems and the interaction of noise with thresholds (Harris & Wolpert, 1998; Beggs & Plenz, 2003; Newell *et al*. 2006; Gold & Shadlen, 2007; Patzelt *et al*. 2007; Beck *et al*. 2008; Carpenter *et al*. 2009; Insperger & Milton, 2014). Some of these investigations are in a context of neuronal models and decision making processes where thresholds are important. However, we observe that when it comes to providing a close model based explanation of experimental data from a sustained sensorimotor control task of the kind represented above (Fig. 1), the explanation of Kleinman *et al*. remains pre‐eminent: The motor control signals in sustained sensorimotor control tasks have been explained most completely and most accurately as a linear process with random noise accounting for the non‐linear remnant (Kleinman, 1969; Levison *et al*. 1969; Kleinman *et al*. 1970; van der Kooij & de Vlugt, 2007; Kiemel *et al*. 2011; van der Kooij & Peterka, 2011).

Despite the success and accessibility of the linear model, there is partial evidence for the view that manual tracking is a serial, refractory process (Navas & Stark, 1968; Miall *et al*. 1986; Loram *et al*. 2011). Recently, it has been demonstrated that sustained manual tracking involves substantial serial, refractoriness related to the order of the controlled system (350 and 550 ms for 1st and 2nd order, respectively) (van de Kamp *et al*. 2013; Loram *et al*. 2014). Since refractoriness is a non‐linear process, this evidence calls for re‐examination of the extent to which *non‐linear operations* can explain the non‐linear remnant without requiring noise. Refractoriness means insensitivity to sensory information for a certain duration. Within a closed loop process such as tracking, a refractory duration implies the feedback loop is open for a corresponding open loop interval. Deciding when to close the feedback loop to sample sensory information requires a trigger process. It has been established previously that biological sampling is triggered by a signal crossing a threshold rather than a regular clock (Navas & Stark, 1968; Miall *et al*. 1986; Gold & Shadlen, 2007; Beck *et al*. 2008; Carpenter *et al*. 2009; Gawthrop *et al*. 2011; Loram *et al*. 2014). Hence the threshold and the trigger signal are the underlying mechanism determining the observed distribution of open loop intervals and corresponding visuo‐motor delays.

In this experiment, we examined the power of the joystick signal from manual control of a dynamic system subject to a periodic multi‐sine input disturbance (Fig. 1). We created conditions designed to vary prediction error thresholds and distributions of open loop intervals in a fashion predictable from previous published work (Loram *et al*. 2009, 2011; van de Kamp *et al*. 2013). Participants controlled two different systems (1st and 2nd order) and used three instructions (minimise position error, minimise velocity error, minimise manual intervention).

Our first question concerns the efficacy of explaining the non‐linear remnant using noise and using thresholds. These explanations are not exclusive, rather one explanation is more specific than the other. Noise provides a general, empirical description of the non‐linear remnant. As explained by Levison *et al*. (1969) the ‘equivalent observation noise’ processes may include such effects as (1) true observation noise, (2) motor noise, (3) random variations in controller gain and time delay, and (4) effects of aperiodic signal sampling by the human. Thresholds, causing aperiodic sampling provide a mechanistic explanation that is more specific with respect to process and possible neural substrate. In our investigation, we consider thresholds on the error in the prediction of the state of the system (position and velocity), rather than on deviation of the state from a set point. Joystick power was analysed using three models: (1) we used the continuous optimal, predictive control model of Kleinman *et al*. (1970) (CC), (2) we used the same continuous optimal, predictive control model with observer noise spectra calculated to fit the remnant (CCN) (Levison *et al*. 1969); and (3) we used the intermittent open loop predictive control model of Gawthrop *et al*. (2011, 2015), with thresholds on the prediction error of the states (IC). The default hypothesis would be that a continuous linear mechanism, with added noise (CCN) provides a better explanation of the complete power spectrum (linear component plus non‐linear remnant) than a wholly deterministic intermittent control mechanism (IC).

Our second question concerns the strength of the evidence that continuous manual tracking depends upon a serial, refractory motor decision making process *within* the sensorimotor feedback loop. To be consistent with existing evidence (Levison *et al*. 1969), a convincing general noise explanation requires addition to the observed states of a noise spectrum which changes smoothly with frequency, such as white or filtered white noise. Between conditions, the noise normalised to the variance of the displayed system output should be independent of system order, controller parameters, disturbance amplitude and bandwidth (Levison *et al*. 1969). Between conditions, a convincing threshold explanation requires thresholds that change plausibly with the instructions. A convincing threshold explanation should also produce a distribution of open loop intervals that relates plausibly to the instructions and with values that increase with system order (van de Kamp *et al*. 2013). The default hypothesis would be that wholly deterministic intermittent control does not meet these conditions.

Our underlying question concerns the physiological mechanisms of visually guided manual control. Experimental evidence for open loop intervals at the level of sub‐movements, on the temporal scale of 200–500 ms, lying *within* the sensorimotor feedback loop, places constraints on the connectivity of those neural substrates supporting threshold related motor decision making to those substrates supporting sensory analysis and initiation of motor output (Frank, 2011; Hardwick *et al*. 2013; Loram *et al*. 2014; Scott *et al*. 2015; Caligiore *et al*. 2017).

## Methods

### Ethical approval

The experiments reported in this study were approved by the Academic Ethics Committee of the Faculty of Science and Engineering, Manchester Metropolitan University and conform to the *Declaration of Helsinki*. Participants gave written, informed consent to the experiment.

### Experimental details

Our experiment replicated the design of Levison *et al*. (1969). We consider the characteristics of remnant obtained from manual control situations in which (1) the plant dynamics are linear, (2) the task requirements are such that the subject apparently devotes continuous attention to the tracking task, and, as a further simplification, (3) the subject manipulates a single control.

Twelve healthy adults (10 male and 2 female, 36 ± 13 years, mean ± SD) used a high precision, sensitive, contactless, uniaxial finger operated joystick (HFX Magnetic, CH Products Ltd, UK) to sustain control of a single input, single output dynamic system (Fig. 1). The output was displayed as the left–right position of a dot on an oscilloscope (Fig. 1). Hence participants had continuous visual feedback of the system output (dot on screen) and continuous haptic feedback of system input (joystick position). Participants controlled each of two previously published systems: an unstable (2nd order) system with an unstable time constant of 0.9 s and no friction or damping and a stable, first order system (Loram *et al*. 2009; van de Kamp *et al*. 2013). For the first order system, the joystick determined velocity of the dot position. For the second order system, the joystick determined acceleration of the dot position. Since the second order system is unstable with a short time constant, this task required attention to the oscilloscope and precise, gentle manual control, mainly of the index finger and thumb, which was acquired easily following prior familiarisation and practice of at least 15 min. Participants were asked to maintain continuous contact with the joystick to ensure that control was as continuous as possible (Loram *et al*. 2011).

The system was disturbed at the input using a specifically constructed multi‐sine signal, containing 100 frequency components of equal amplitude, equally spaced over the range 0.1–10 Hz. For each trial the phases were randomised and the crest factor (ratio of maximum deviation to SD) was limited to 3 making the signal unpredictable but periodic (Pintelon & Schoukens, 2001). The amplitude of the disturbance was chosen to be large enough to allow analysis, but no larger than necessary so as to keep conditions close to the undisturbed conditions used in previous studies of pursuit tracking (van de Kamp *et al*. 2013).

For the first order system, we investigated one instruction.

‘1st’: ‘keep the dot as close to the centre as possible’ (minimising position error),

For the second order system, we investigated three instructions

‘pos’: ‘keep the dot as close to the centre as possible’ (minimising position error),
‘vel’: ‘keep the dot as still as possible, but don't care where it is’ (minimising velocity error), and
‘mi’: ‘while keeping the dot on screen, wait as long as possible before intervening’ (minimising intervention).

The purpose of the last condition (mi) was to ensure that the control was explicitly related to a position threshold making it non‐continuous. To conform with this instruction, participants had to wait for the dot to approach the edge of the screen before using the joystick to bring it under control. The four conditions (1st, pos, vel, mi) were presented in random order and each trial lasted for 200 s. Participants were offered a break of 5 min between trials.

The dynamic system was implemented in Simulink and executed using the Real Time Workshop (Matlab v7, The Mathworks, USA) on a laptop PC. The joystick signal (*u*_e_) and the oscilloscope output (corresponding to system output *y*) were interfaced via a data‐acquisition card (DAQ card 6036E, National Instruments, USA) at a sample rate of 1 kHz to 16 bit precision. Recorded signals (disturbance *d* and joystick signal *u*_e_, cf. Fig. 1) were downsampled to 100 Hz.

### Analysis procedures

#### Calculation of experimental frequency response at excited and non‐excited frequencies

Throughout this paper frequency response describes the relationship between joystick (*u*_e_) and disturbance (*d*). The injected disturbance signal, *d*, contains components only at 100 discrete frequencies (0.1, 0.2, …, 10 Hz). The frequency response can be obtained at these excited frequencies. The response at intermediate 100 non‐excited frequencies (0.05, 0.15, 9.95 Hz), where no disturbance is applied, is considered the remnant response. The frequency response at excited and non‐excited frequencies can be calculated by analysing periods of 20 s.

#### Overview: model based fitting of experimental frequency response

The fitting of models proceeds in two stages. A detailed description of the models is given in Supporting information.

The mean complex frequency response (magnitude and phase) defines the linear, periodic component of the response (Pintelon & Schoukens, 2001). In stage I, the mean, complex frequency response at excited frequencies is used to estimate the controller parameters and time delay appropriate for the mean response (Table 1).

**Table 1:** Controller design parameters (mean ± SD)

		1st	pos	vel	mi
*Q*_c_	CC	27.5 ± 61.9	3.9 ± 5.25	3.56 ± 4.59	258 ± 873
		25.3 ± 45.4	10 ± 27.8	0.313 ± 0.695	(0.641 ± 2.17) × 10^3^
		140 ± 330	9.17 ± 28.3	0.0116 ± 0.04	(1.75 ± 3.86) × 10^3^
		—	1.38 ± 4.77	4.3 ± 10.1	3.02 ± 8.1
	IC	29.7 ± 59.2	3.96 ± 4.94	4.11 ± 4.37	258 ± 873
		127 ± 396	11 ± 27.9	0.209 ± 0.538	(0.642 ± 2.17) × 10^3^
		(0.894 ± 2.87) × 10^3^	9.26 ± 28.3	1.8 ± 2.91	(1.75 ± 3.86) × 10^3^
		—	1.73 ± 5.7	5.2 ± 10	3.74 ± 7.87
$q_{s}$	CC	45 ± 69.8	5.87 ± 14.5	1.3 ± 1.44	0.504 ± 0.812
	IC	45.4 ± 70.3	5.87 ± 13.6	1.48 ± 1.43	1.23 ± 1.65
*t*_d_(ms)	CC	111 ± 21.1	187 ± 20.3	174 ± 18	206 ± 48.1
	IC	50	50	50	50
Δ_s_(ms)	IC	95.3 ± 79.9	134 ± 19.3	111 ± 27.4	152 ± 39.2

Mean delays (ms): td(1 st  order )=111±21.1, td(2 nd  order )=189±33.8. Mean sampling delays (ms): Δs(1 st  order )=95.3±79.9, Δs(2 nd  order )=132±33.7. *Q*_c_ values are the LQR controller design weights for the states, and *q*_o_ is the relative weight for the LQR observer design weights, i.e. *Q*_o_ = *q*_o_ × *Q*_c_. *t*_d_ is the loop delay, Δ_s_ denotes the sampling delay. Parameters for the continuous controllers (CC and CCN) are the same and denoted by CC, while the parameters for the intermittent controller are denoted by IC.

The power spectrum at non‐excited frequencies represents the non‐linear remnant alone. At excited frequencies, the power includes a linear component and a non‐linear component (Pintelon & Schoukens, 2001). The power spectrum at excited and non‐excited frequencies contains all the power generated. In stage II, the power spectral density (PSD) at excited and non‐excited frequencies is used to fit two alternative explanations. In the first general descriptive explanation we estimate the noise parameters for the continuous model. In the second, more specific mechanistic explanation we estimate the threshold parameters for the intermittent control model.

#### Stage I: identification of controller parameters (CC)

**Figure 2:**
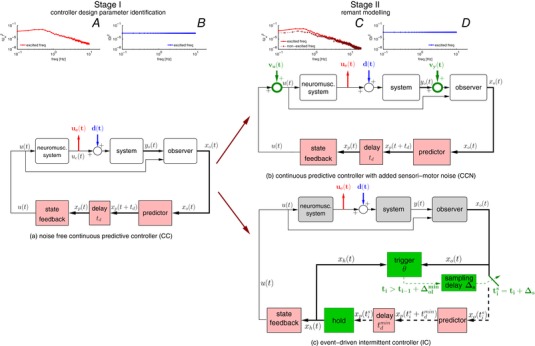
Hypothesis and methodology In stage I (left side of the figure) controller design parameters are identified for a noise‐free predictive controller (diagram (*a*)) by fitting the frequency response function between *U_e_* (panel *A*) and *D* (panel *B*) at excited frequencies (i.e. |*D*| > 0) (obtained from time‐domain data shown in Fig. 1 *A* and *B*). In stage II (right side) the remnant component is considered by increasing the analysis period of the data (panels *C* and *D*). The controller design parameters from stage I are used with two alternative explanations for remand (highlighted in *B* and *C*): a predictive controller with suitably coloured motor (*v_u_*) or sensory (*v_y_*) noise (diagram (*b*)), and event‐driven intermittent control with appropriately selected prediction error thresholds (diagram (*c*)). [Color figure can be viewed at wileyonlinelibrary.com]

The intermittent control model generates predicted open loop control signals *x*_h_ (Fig. 2 *C*) using the same control parameters as the underlying continuous controller (Gawthrop & Wang, 2011; Gawthrop *et al*. 2011, 2015). Hence the same controller parameters are appropriate for both continuous and intermittent control models (Gollee *et al*. 2012). In stage I, a noise‐free, optimal, linear continuous‐time predictive controller (CC) is fitted at the excited frequencies to the complex frequency response function (Fig. 2 *A*). Taking advantage of the periodic disturbance to produce an exact frequency analysis with no leakage (Pintelon & Schoukens, 2001), we calculated the mean complex frequency response using non‐overlapping periods of 10 s and no window (Halliday *et al*. 1995). This complex frequency response contains all the magnitude and phase information available for estimating mean time delays and controller parameters. For each trial, the controller design parameters, i.e. linear‐quadratic weightings for state‐feedback gain *Q*_c_, observer gain *Q*_o_, and mean time‐delay, *t*_d_ were optimised to minimise mean square error between complex model and experimental values of the frequency response (Table 1).

#### Stage II: fitting the complete power spectral density, including non‐linear remnant (CCN, IC)

In stage II, the individual controller design parameters obtained in stage I, were used to model the complete mean power spectral density (PSD) at all excited and non‐excited frequencies (Table 2). The PSD at excited and non‐excited frequencies includes all the power within the signal, including power unrelated or related inconsistently to the input disturbance whereas the mean complex frequency response underestimates the total power on account of cancellation through averaging periods with variable phase. With increasing number of periods, the mean PSD provides an increasingly reliable estimate of the total power at all frequencies. Two approaches were considered to estimate the total power including the remnant response: (1) adding measurement (sensory) noise, *v_y_* to the continuous controller from stage I (CCN), (Fig. 2 *B*), or (2) using an intermittent controller with prediction error thresholds and without added noise (IC), (Fig. 2 *C*). The same cost function (*J*) was used to evaluate all explanations (Table 3) where *N* is the number of excited and non‐excited frequencies, *U*_e_ is the power of the joystick signal, ω is frequency and *j* is the unit imaginary number.

(1) $$J\;=\frac{1}{N_{\omega }}\;\;\sum_{\omega }({|U_{e}\left(j\omega \right)|}^{2}-{|\hat{U_{e}}\left(j\omega \right)|}^{2}).$$

(1) *Noise explanation (CCN)*. A continuous‐feedback control model is used (Fig. 2 *B*). Within the frequency domain, the noise spectrum *V_y_* is calculated using the measured joystick signal *U*_e_ at the 100 non‐excited frequencies and, using the corresponding loop transfer function given by:
  (2) UeVy=CP NMS 1+L,

where *C*, *P*_NMS_ and *L* are, respectively, the controller, neuromuscular system and loop gain functions (Levison *et al*. 1969). The calculated noise signal is also applied at the excited frequencies by interpolating between neighbouring non‐excited frequencies. Note that, apart from the added noise, this model (CCN) is identical to that obtained in stage I (CC).

(2) *Threshold explanation (IC)*. A noise‐free intermittent controller is considered (Fig. 2 *C*).

**Table 2:** Normalised simulated power (mean ± SD)

*P*_n_ (%)	1st	pos	vel	mi
CC	48.4 ± 12.5	35.9 ± 13.9	35.1 ± 13.5	8.1 ± 5.3
CCN	96.7 ± 8.8	98.8 ± 5.9	98.3 ± 6.0	94.9 ± 7.2
IC	77.1 ± 14.1	85.4 ± 17.8	87.6 ± 13.5	87.8 ± 17.8

Total simulated power normalised by the corresponding total experimental power.

**Table 3:** Cost function values (mean ± SD)

*J *× 10^−5^	1st	pos	vel	mi
CC	0.48 ± 0.55	2.9 ± 3.7	1.7 ± 0.94	15 ± 12
CCN	0.067 ± 0.037	0.32 ± 0.4	0.2 ± 0.15	0.92 ± 0.76
IC	0.15 ± 0.13	0.64 ± 0.66	0.43 ± 0.31	1.4 ± 0.78

The intermittent control model is based upon the continuous control model (Fig. 2 *B*) but includes three additional processes shown in green (Fig. 2 *C*): a trigger, a sampling switch, and a hold. The hold uses a model of the continuous closed loop system to generate a predicted, open loop control signal. In the absence of disturbances and measurement noise, this control signal is identical to that produced by the continuous controller. The trigger uses prediction error, which is the difference between the continuous predicted and observed states, to decide when to instantaneously close the switch to sample the states to update the hold process. The trigger has three parameters, including velocity and position prediction error thresholds (θ^vel^, θ^pos^), and a sampling delay ∆_s_. Sampling is triggered when the mean square prediction error exceeds an ellipse defined by the velocity and position prediction error thresholds (θ^vel^, θ^pos^) and also when a minimum open loop interval ∆_ol_^min^ = *t*_d_ *+ *∆_s_, is exceeded. The time delay *t*_d_ provides a fixed minimum transport delay.

The sampling delay ∆_s_ provides a variable component to the minimum open loop interval. The sampling delay enforces a maximum speed–minimum accuracy limit since it defines the minimum time for the observer to settle before sampling system states (Gawthrop *et al*. 2015). When the sampling delay is small, triggering is determined by the thresholds and is typically aperiodic. When the sampling delay is large, and/or the thresholds are small, triggering is more regular determined primarily by the minimum open loop interval.

**Figure 3:**
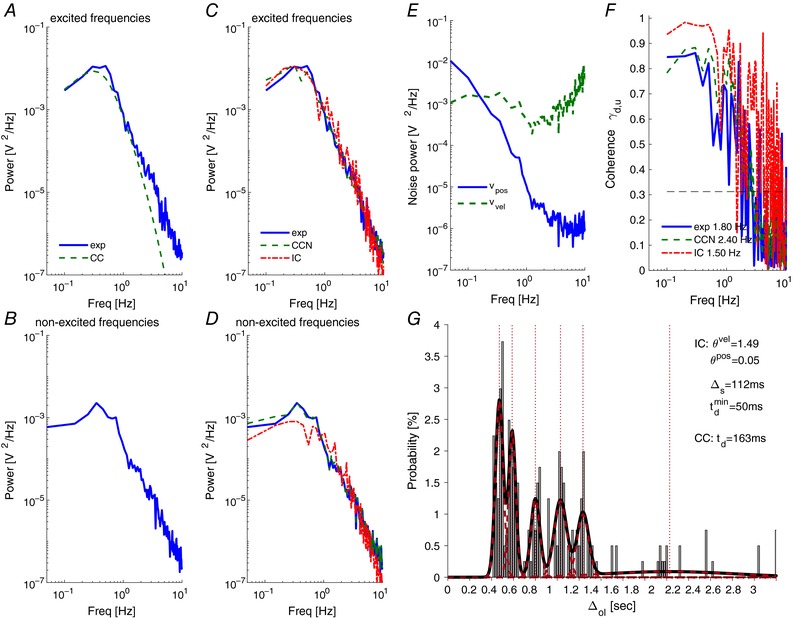
Representative individual result, control instruction ‘pos’ Panels *A* and *B* show the power spectra estimated from experimental data (thick line), together with those from the best fit approximations with a noise‐free continuous predictive controller (dashed line) at excited frequencies (*A*) and non‐excited frequencies (*B*). Panels *C* and *D* show the corresponding spectra (1) for a CCN with suitable added coloured noise *v_y_* (observation noise spectrum shown in panel *E*), and (2) for an event‐driven intermittent controller with adjusted thresholds. The threshold values and the resulting distribution of the intermittent intervals ∆_ol_ are shown in panel *G*, together with an approximation by six Gaussian distributions (forming a Gaussian mixture model (GMM), centres indicated by dotted lines). Panel *F* shows the coherence *γ_uy_* for experimental data and the event‐driven IC, together with the estimated coherence limits crossing the threshold of significance (horizontal dashed line). [Color figure can be viewed at wileyonlinelibrary.com]

The LQR design parameters obtained in stage I were used as the initial design parameters, and the time delay set to a small fixed value of *t*_d_^min^ = 50 ms (representing a minimal physiological transport delay). Using simulation to produce model based power spectra, the parameters θ^vel^, θ^pos^ and ∆_s_ were initially optimised and then also the LQR design parameters were re‐tuned to minimise the mean square error between model and experimental power at all frequencies (excited and non‐excited). This threshold model produced a distribution of open loop intervals (Fig. 3 *E*).

#### Summary of quantities calculated from experimental and fitted simulation data

The following were calculated for individual trials during stage II.

‐ Power spectra of the joystick signal at excited and non‐excited frequencies (Fig. 3 *A*–*D*).

For the noise explanation (CCN, Fig. 2 *B*) we calculated:

‐ Sensory noise power spectra (*V_y_*) added to measurements of velocity and position (Fig. 3 *E*).
‐ Normalised noise power spectra, calculated by dividing *V_y_* by the variance of the system position (*y*) (Levison *et al*. 1969).
‐ Normalised noise power spectra, calculated by dividing *V_y_* by the variance of the joystick signal (*u*_e_).
‐ Coherence (*γ_uy_*) between disturbance (*d*), and joystick signal (*u*_e_) at excited frequencies (Fig. 3 *F*; note that the coherence is equal to 1 at all frequencies for the continuous‐feedback controllers CC).
‐ Coherence limit, i.e. the smallest frequency at which significance at 95% confidence was first lost (Figs 3 *F* and 5 *D*).

For the threshold explanation (IC, Fig. 2 *C*) we calculated the following:

‐ Coherence (*γ_uy_*) and Coherence limit as above.
‐ Prediction error thresholds for position and velocity (Figs 3 *G* and 5 *B* and *C*).
‐ Distribution of intermittent (open loop) intervals *(∆*_ol_) (Fig. 3 *G*).
‐ Gaussian mixture model (GMM) decomposition of intermittent interval distribution (Fig. 3 *G*; the distribution was approximated by a series of weighted normal Gaussian distributions (McLachlan & Peel, 2000)).

### Statistical analysis

For statistical analysis of changes in noise spectrum between instructions for 2nd order systems at alpha 0.05, we used 1‐d statistical parametric mapping (SPM‐1D). SPM‐1D is now well established as the appropriate method for comparing vector quantities (e.g. power at multiple frequencies) rather than scalar quantities (e.g. power at one frequency) (Pataky *et al*. 2013). SPM‐1D calculates the chosen statistic at each frequency and calculates a threshold of significance appropriate for ordinally sampled measurements of partial independence. SPM‐1D avoids the false positives of multiple scalar tests and avoids the false negatives of scalar tests with Bonferroni correction. We used the freely available implementation (www.spm1d.org, Matlab 2015*b*, Pataky, 2012) to calculate the *F* statistic for with repeated measures ANOVA (*N* = 36, 3 levels) followed by *post hoc* pairwise *t* tests.

## Results

For this manual tracking task, our analysis concerns the efficacy of three explanations (CC) continuous linear feedback alone, (CCN) continuous feedback with general sensorimotor noise, and (IC) aperiodic intermittent feedback at times determined by prediction error thresholds.

### The linear response at excited frequencies (Stage I)

In analysis stage I (CC, Fig. 2 *A*) we identified the parameters of a linear, continuous controller (CC) that gives a best least squares fit at the frequencies excited by the disturbance signal (Table 1). The feedback delays for 1st and 2nd order systems at 111 ± 21 ms and 189 ± 34 ms, respectively (Table 1), were consistent with model independent, experimental values published previously (Loram *et al*. 2009).

**Figure 4:**
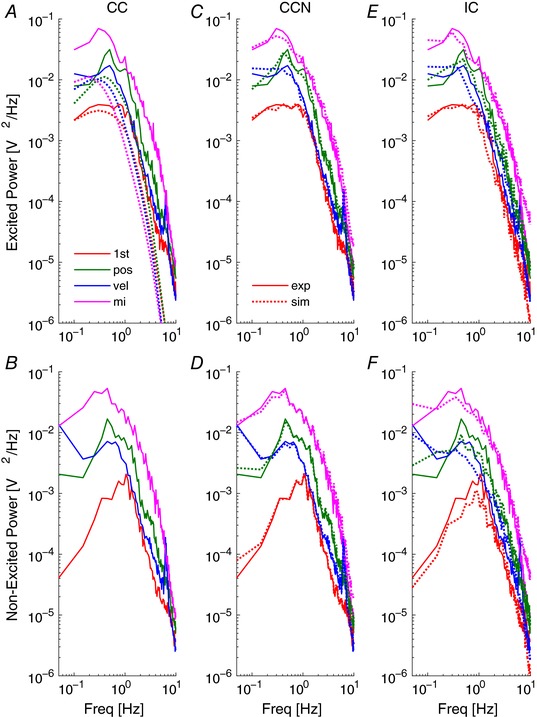
Explanations of human joystick power using continuous and intermittent control models *A*, *C* and *E*, mean power spectra at excited frequencies. *B, D* and *F*, mean power spectra and non‐excited frequencies. Experimental data (continuous lines), simulations from fitted models (dotted lines). Columns show model fits from noise‐free linear continuous control (CC), continuous control with added noise (CCN) and intermittent control with aperiodic sampling (IC). Results are shown for the 1st order system (1st) and the three different instructions for the 2nd order system (pos, position control; vel, velocity control; mi, minimise intervention). Panels summarise all 12 participants. [Color figure can be viewed at wileyonlinelibrary.com]

Typically, CC fits experimental power well at low frequencies, and under‐represents experimental power at higher frequencies (Fig. 3 *A*). Without added noise, relatively little of the total power is explained by the linear controller (Fig. 4 *A*). As a percentage of total experimental power, the power explained by CC at excited frequencies is: 48.2 ± 12.4%, 35.9 ± 13.9%, 35.1 ± 13.5%, and 8.1 ± 5.3% (mean ± SD) for 1st, pos, vel and mi conditions, respectively (Table 2). A linear controller produces no power at non‐excited frequencies (Fig. 4 *B*).

### Fitting the non‐linear remnant (Stage II)

#### Noise explanation (CCN, Fig. 2 *B*)

We computed the noise spectrum (*v_y_*, Fig. 3 *E*) required to replicate the experimental power observed at non‐excited frequencies (e.g. Fig. 3 *C*), and applied this at all frequencies. Simulating data using the computed control parameters and noise signal, it is no surprise that experimental power at non‐excited frequencies is reproduced for the representative participant (Fig. 3 *D*) and generally for the whole group in all conditions (Fig. 4 *C* and *D*). We note that with the added noise, simulated power at excited frequencies also fits well the experimental power (Fig. 3 *C vs*. 3*A* and Fig. 4 *C vs*. 4*A*) and coherence (Fig. 3 *F*) across the entire frequency range. According to this explanation, some of the power at excited frequencies, and all of the power at non‐excited frequencies, originate from noise. As a percentage of total experimental power, the power explained by CCN is 96.4 ± 8.8%, 98.7 ± 6.0%, 98.2 ± 5.9%, and 94.4 ± 6.8%, (mean ± SD) for 1st, pos, vel and mi conditions, respectively (Table 2). The noise model used for this has 100 complex parameters (one at each non‐excited frequency).

#### Threshold explanation (IC, Fig. 2 *C*)

IC with adjusted prediction error thresholds replicates experimental power at excited frequencies (Fig. 3 *C*) and at non‐excited frequencies for the representative participant (Fig. 3 *D*). Typically, the distribution of open loop intervals includes substantial durations of 0.3 s and longer (Fig. 3 *G*). Sampling at these intervals is associated with reduced coherence between disturbance and control signal beyond 1–2 Hz (Fig. 3 *F*).

This deterministic model, with two adjusted thresholds and sampling delay reproduced power well at excited and non‐excited frequencies across all participants in all conditions (Fig. 4 *E* and *F*). As a percentage of total experimental power, the power explained by IC is 76.9 ± 14.1%, 85.4 ± 17.9%, 87.5 ± 13.5%, and 87.3 ± 17.7%, (mean ± SD) for 1st, pos, vel and mi conditions, respectively (Table 2). In contrast to the 100 complex parameters of the CCN model, the IC explanation only required three parameters.

### Comparison of explanations: overview

Using the same cost function for all explanations, Table 3 shows that the linear continuous controller (CC) provided the least effective fit to experimental power. Adding noise to the continuous controller reduced the error by a factor of 7 to 16 (CCN). The wholly deterministic model (IC) reduced the error from CC by a factor of 3 to 10. The improved fit, by adding noise or using thresholds, was most marked for the minimise intervention (mi) condition (factors 16 and 10, respectively).

### Comparison of explanations: difference between conditions (1st, pos, vel, mi)

#### Noise explanation (CCN)

**Figure 5:**
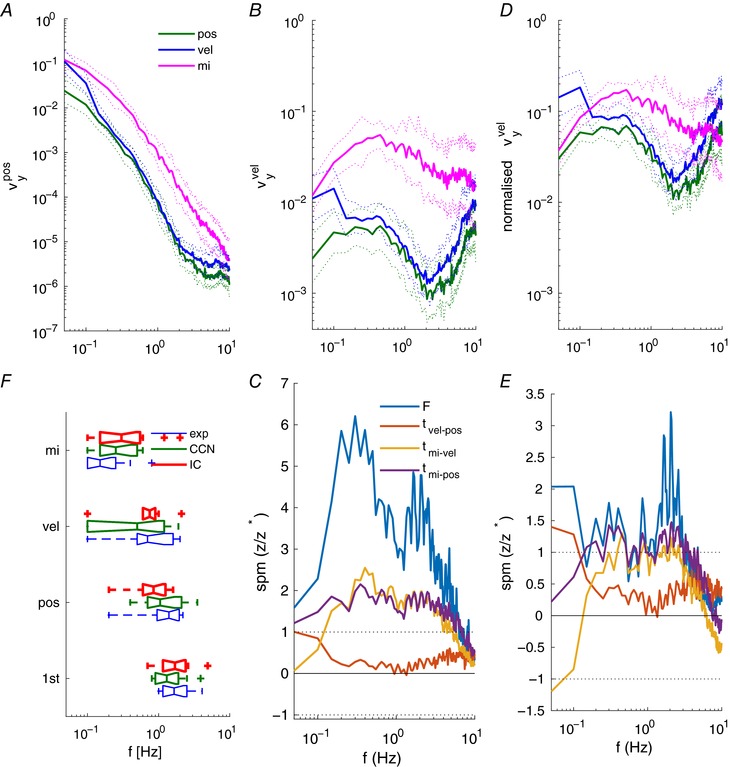
Equivalent observation noise required to explain the non‐linear remnant *A* and *B*, equivalent observation noise added respectively to position and velocity states. Values are mean ± 95% confidence interval for each condition (pos, vel, mi). *D*, normalised equivalent observation noise (mean ± 95% ci). Noise to added velocity state divided by variance of velocity signal (Levison *et al*. 1969). *C* and *E*, temporal evolution of SPM‐1d test statistic relative to threshold of significance. The *F* statistic (blue) reports the repeated measures ANOVA test for difference between conditions (pos, vel, mi). The *t* statistics report pairwise comparisons between conditions (pos, vel, mi). *F*, coherence limits for experimental data, and matched model simulations (CCN, IC). These values represent the lowest frequency at which coherence changes from significant to non‐significant. Panels summarise all 12 participants. [Color figure can be viewed at wileyonlinelibrary.com]

For all 2nd order conditions, Fig. 5 *A* and *B* shows the amplitude of observation noise at all frequencies required to fit the manual power spectrum. The required observation noise spectrum reflected onto the velocity state is closest to ‘white noise’, i.e. constant amplitude at all frequencies (Fig. 5 *B*). The required spectrum reflected onto the position state is closest to ‘1/*f*’ noise (Fig. 5 *A*), and is the same values as Fig. 5 *B* transformed by unity and 1/(2π*f*)^2^ for 1st (not shown) and second order systems respectively. Between instructions for 2nd order systems, there were statistically significant changes in noise at all frequencies up to 8 Hz (Fig. 5 *C*). *Post hoc* pairwise testing confirmed statistically that pairwise changes were distributed non‐uniformly within the frequency domain. The change of instruction from ‘keep the dot close to the centre’ to ‘keep the dot still’ (i.e. ‘pos’ to ‘vel’, Fig. 5 *B*) resulted in a 4‐fold increased observation noise significant statistically below 0.1 Hz with little change in noise above 0.3 Hz. The change of instruction from ‘keep the dot still’ to ‘minimise intervention’ (i.e. ‘vel’ to ‘mi’, Fig. 5 *A* and *B*) resulted in an order of magnitude increased noise significant statistically above but not below 0.15 Hz.

Figure 5 *D* shows the signal‐normalised observation noise presented for the velocity state consistent with Levison *et al*. (1969). For 2nd order systems, between instructions there were significant changes in signal‐normalised noise up to 4 Hz (Fig. 5 *E*). Signal‐normalised noise showed significant variation in spectral shape including a 5‐fold increase in normalised noise below 0.1 Hz (pos→vel) and up to an order of magnitude increase above 0.2 Hz (pos→mi) (Fig. 5 *E*). Finally, between conditions, particularly system order, the added noise replicates the experimental changes in the frequency limit of significant coherence (Fig. 5 *F*).

#### Prediction error threshold explanation (IC)

For all conditions, effects of instruction and system order are associated with changes in the trigger parameters (thresholds θ^vel^, θ^pos^, and sampling delay ∆_s_ *)*.

For the first order system, the position prediction error threshold is very low and the sampling delay relatively long (95 ± 80 ms; Table 1), close to the lower limit for the distribution of open loop intervals. The distribution of open loop intervals is relatively narrow showing maximum probability in the range 300–350 ms and negligible open loop intervals less than 100 ms.

**Figure 6:**
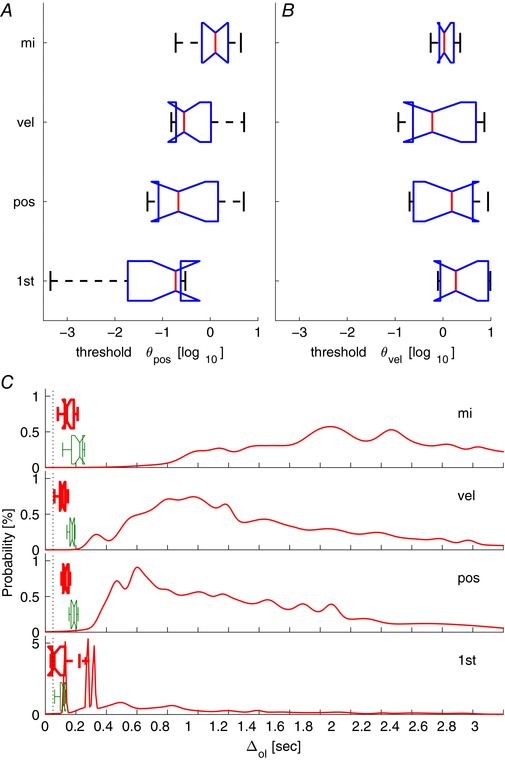
Explanation of linear power and non‐linear remnant using intermittent control with aperiodic sampling *A* and *B*, prediction error thresholds for velocity (θ^vel^) and position (θ^pos^) states. *C*, distributions of centres of GMMs approximating the intermittent intervals distributions (continuous line). Sampling delay used in IC (red box). Constant delay used in CC and CCN (green box). Panels summarise all 12 participants, for the four conditions (1st, pos, vel, mi). [Color figure can be viewed at wileyonlinelibrary.com]

For the second order systems, the collective sampling delay is 132 ± 34 ms (Table 1), and considerably below the lower limit of the distribution of open loop intervals (Fig. 6 *C*). The change of instruction from ‘minimise position error’ (pos) to ‘minimise velocity without caring about position’ (vel), was associated with reduced prediction error threshold for velocity (Fig. 6 *B*), with decreased sampling delay (Table 1), and a shift from a broad distribution of open loop intervals centred around 550–600 ms to one centred around 800–1000 ms (Fig. 6 *C*). Both distributions show negligible open loop intervals below 200 ms. The change of instruction from ‘minimise velocity’ (vel) to ‘minimise intervention while remaining within the bounds of the screen’ (mi), was associated with increased prediction error threshold for position (Fig. 6 *A*), increased sampling delay, and a shift in the distribution of open loop intervals to a broader distribution with higher central values around 1800–2200 ms (Fig. 6 *C*). There were negligible open loop intervals below 300 ms.

Between conditions, particularly system order, the IC simulations replicate the experimental changes in the frequency limit of significant coherence (Fig. 5 *F*). For all conditions the constant delay identified for the linear continuous controller (CC) lies close to the lower limit of the distributions of open loop intervals (IC) (Fig. 6 *C*).

## Discussion

We examined manual joystick control of a dynamic system subject to a periodic multi‐sine input disturbance (Fig. 1). Relative to the disturbance, the total joystick power contains a linear component and a non‐linear remnant. Our question lies at two levels. At one level we consider the merits of explaining the total joystick power using (1) a linear continuous mechanism with equivalent observation noise (CCN) or (2) an intermittent control mechanism with aperiodic sampling (IC). At a deeper level, our question concerns the prevalence and hierarchical level of motor decision making required while implementing sensorimotor control. Specifically, does a serial, motor decision making process lie *within* the sensorimotor feedback loop implementing manual tracking?

The first explanation (CCN) is a general empirical description which covers four possible physical sources of the non‐linear remnant (motor noise, sensory noise, aperiodic sampling, time varying control) (Levison *et al*. 1969). The second explanation (IC) is more specific in terms of process (one out of four possible), more specific in terms of possible neural substrate, and is wholly deterministic, i.e. the output can be reconstructed from the input using a non‐linear rule. These two explanations are not in competition with each other. The first, general explanation provides a context within which the second, more specific explanation can be considered. The main question and result of this paper is the extent to which the deterministic IC mechanism with aperiodic sampling can explain the human control action.

### The extent to which a linear mechanism alone explains the human operator

During stage I, we fitted a linear continuous model to the periodic portion of the joystick signal (CC, Fig. 2 *A*). The linear continuous model used optimal state feedback, an observer, a predictor and a neuromuscular system. The observer and predictor use prior knowledge of the input–output mapping of the dynamic system, and measurement of sensory information to estimate the current and future states of the system (position, velocity) and the joystick. Following a well‐established engineering and sensorimotor control literature, we understand this model is uncontroversial and provides the best available linear description of the human operator (Kleinman, 1969; Kleinman *et al*. 1970; Bays & Wolpert, 2007; Gawthrop *et al*. 2009; van der Kooij & Peterka, 2011). For second order systems, a most striking point is how little of the joystick power is linear (Fig. 4 *A* and *B*). The power explained by the linear mechanism was at best 48.2 ± 12.4% for the 1st order system and was lowest at 8.1 ± 5.3% for the 2nd order, minimize‐intervention condition. The power, shown in Fig. 4 *A* and *B* not fitted by the linear model (CC), is non‐linear remnant at both excited and non‐excited frequencies. We conclude that, in general, a linear mechanism does not capture the essence of the manual tracking process. This conclusion is consistent with the previous observation that continuous control is neither necessary, nor most effective for these tasks (Loram *et al*. 2011).

#### The use of equivalent observation noise to enable a linear mechanism to explain the human operator

We followed the experimental and analysis methodology of Levison *et al*. (1969) to calculate the noise spectrum that fits best the excited and non‐excited frequencies of the manual power spectrum. This analysis does not test the descriptive ability of this explanation (CCN), since a good fit is inevitable. Rather, this analysis enables us to use our instructions (pos, vel, mi) to produce noise spectra which test previous predictions regarding the noise‐signal relationship (Levison *et al*. 1969).

Levinson *et al*. (1969) reported, that when normalised to the variance of the system position (dot on the screen), the noise spectra were smooth with frequency and were invariant in amplitude and shape with respect to order of the dynamic system (zero, 1st, 2nd), controller parameters, disturbance amplitude and disturbance bandwidth. When reflected onto the observed position and velocity states the noise spectra were 1st order (‘1/*f*’) and ‘white’ respectively. These authors concluded the absolute amount of remnant power scales along with the other signals circulating throughout the system. The tendency of the random component of the human's response to increase with the magnitude of the desired response had already been observed in other investigations not involving manual control and this tendency formed the basis of many subsequent models of controller remnant (e.g. Harris & Wolpert, 1998; van der Kooij & Peterka, 2011).

Levison *et al*. did not study the effect of instruction on altering the control priority of the operator. Our experiment showed that between conditions when instructions changed (pos → vel → mi) the absolute remnant power increased up to 4‐fold and these changes departed from the ‘1/*f*’ and ‘white’ spectral shape by up to an order of magnitude (Fig. 5 *A* and *B*). When normalised to the output signal (Fig. 5 *D*), we found the remnant amplitude was related to the instructed control priority. Relaxation of the constraint to regulate position to the centre (pos → vel) was associated with a substantial increase in low frequency normalised remnant. Relaxation of priority to regulate position to within the screen boundaries with minimum intervention (pos → mi) also resulted in a substantial increase in middle frequency normalised remnant. The effect of control priority was to reduce the noise relative to the signal and hence to make the visuo‐manual control more linear. It appears that linearity is an adjustable consequence of control vigilance.

Since equivalent observation noise and normalised observation noise changed meaningfully with the control priority of the operator, and since equivalent observation noise accounted for a substantial fraction of the total joystick power (64–92% for 2nd order systems) we conclude the non‐linear source of power is intrinsic to the physiological control mechanism.

### Physiological sources of non‐linear remnant

Levison *et al*., and others subsequently, have concluded the source of ‘equivalent observation noise’ lies within the ‘central processor’ of the sensorimotor loop effecting sustained control; however, they did not attempt to pinpoint the physiological source beyond identifying the physical alternatives which included true observation noise, motor noise, aperiodic sampling and changes in controller through time (Levison *et al*. 1969; Harris & Wolpert, 1998).

Voluntary muscle activation produces variability in force that scales with the level of force (Fuglevand *et al*. 1993; Jones *et al*. 2002; Hamilton *et al*. 2004; Dideriksen *et al*. 2012) and approximates typically a ‘1/*f*’ power law spanning the muscle bandwidth (0–20 Hz) (Newell *et al*. 2006). This ‘motor noise’ arises from the orderly recruitment and firing rate variability within the motor neuron pool innervating muscles (Jones *et al*. 2002) and is considered a physiological byproduct of motor output. Sensory cells (e.g. vestibular organs, muscle spindles) also produce variable firing rates with a broadband power law such that neural averaging of ensembles of cells is required to extract meaningful signals (Prochazka, 2000). Changes in the motor signals or the sensory signals would cause broadband noise, proportionate to the signals as an inevitable byproduct of these biological signals.

Our results and analysis (CCN) showed that between conditions the equivalent observation noise showed changes deviating from broadband signal dependent noise. These changes are not characteristic of a broadband physical noise source such as motor noise or sensory noise, and cannot be explained by changes in the amplitude of such noise or of the signals causing them. The non‐smooth changes in equivalent observation noise must come from some other physical source.

In addition to these physiological sources, variability in motor output arises from threshold related processes within the central nervous system. For example, neuronal avalanches occur in complex networks of cortical neurons organised into a critical state (Beggs & Plenz, 2003). Decision making is related typically to the accumulation of multiple noisy sources until one winning source crosses a threshold (Cisek & Kalaska, 2005; Gold & Shadlen, 2007). Reaction times, errors, and ramping activity of neurons in the posterior parietal cortex are captured by Bayesian sequential sampling models of the accumulation of sensory evidence about stimulus identity (Beck *et al*. 2008; Carpenter *et al*. 2009). The subthalamic nucleus (STN), the major node of the hyperdirect pathway from frontal cortex to STN to basal ganglia output, regulates the time taken to select between alternative competing potential actions (Frank, 2011). Typically, the STN increases the time taken to respond to prevent impulsive decisions, by dynamically (and transiently) adjusting decision thresholds as options are being considered (Cohen & Frank, 2009). For example, the posterior lateral prefrontal cortex is one known central bottleneck of amodal information processing that exhibits serial queuing of response selection activity under dual‐task conditions (Dux *et al*. 2006).

Between conditions, our results and analysis (IC) showed thresholds and distributions of open loop intervals consistent with instructions. We conclude these threshold related processes, and neural substrates listed above, are a likely source of the non‐linear remnant.

### Explanation of the processes modelled by intermittent control with aperiodic sampling

Our wholly deterministic explanation (IC) uses the previously published intermittent control model, with prediction error thresholds and sampling delay to generate aperiodic sampling of system states (position, velocity, joystick) (Gawthrop & Wang, 2011; Gawthrop *et al*. 2011, 2015). This intermittent control model (IC) is based upon the same continuous control model above (CC), and uses two additional processes, a *hold* and a *trigger‐switch* (Fig. 2 *C*). The hold uses a known mapping of the input–output relationship for the underlying continuous closed loop system. Given a current sample of all system states, it generates an open loop, time evolving, control signal which, in the absence of disturbances or measurement errors, is identical to that generated by the underlying continuous, linear controller. The trigger‐switch uses prediction error thresholds and a sampling delay to decide when to close the feedback loop and take a new sample of sensory information. This mechanism uses three parameters, two prediction error thresholds (θ^vel^, θ^pos^), and a sampling delay Δ_s_ to replace the 100 parameters (one amplitude for each non‐excited frequency) used in the noise spectrum analysis. Since the transport delay *t*_d_ was fixed at a very low value (50 ms), the three sampling parameters are free to provide an alternative explanation of visuo‐motor delay in which the fixed delay of the continuous system and added noise is replaced by an aperiodic distribution of open loop intervals plus the irreducible physiological transport delay of 50 ms. Because these are event related durations, not transport delays, the effective stimulus–response delay of the ‘sampling delay’ and an ‘open loop interval’ is equal to half the ‘sampling delay’ and half the ‘open loop interval’ respectively.

Below we show these results are consistent with previous measured, model independent values.

Independent of any model based explanation, control of the first order system is known to have a short feedback loop delay (120 ± 20 ms), small non‐linear component (Loram *et al*. 2009) and moderate refractoriness in pursuit tracking (350 ms) (van de Kamp *et al*. 2013). Consistent with these independent results, the IC model fit of a higher sampling delay of 95 ± 80 ms (Fig. 6 *C*) and low position prediction error threshold (Fig. 6 *A* and *B*) resulted in a narrow distribution of sampling intervals centred around 300–350 ms (Fig. 6 *C*). The lower limit of open loop intervals is consistent with the fixed delay estimated for the continuous controller (CC) in stage I (110 ± 20 ms; Table 1; Fig. 6 *C*) and previously (Loram *et al*. 2009).

For the second order system, measurements independent of any model based explanation have demonstrated a longer feedback loop delay (180–220 ms), a larger non‐linear component (Loram *et al*. 2009) and more substantial refractoriness during pursuit tracking (550 ms) (van de Kamp *et al*. 2013). Our model based analysis here (IC), compared with the first order system, revealed a larger sampling delay (Fig. 6 *C*) and larger thresholds (Fig. 6 *A* and *B*) consistent with less regular triggering and a wider distribution of open loop intervals (Fig. 6 *C*). The distribution of open loop intervals has a lower limit around 200 ms close to the fixed delay estimated with linear continuous control (CC; Table 1) and previously. When position was regulated (pos) the broad distribution of open loop intervals (200–3000 ms) had a central tendency around 550–600 ms. This distribution of open loop intervals is consistent with the range of reaction times we observed during control of a second order system for pursuit tracking (van de Kamp *et al*. 2013). This central value concurs with the refractory duration estimated previously.

Between conditions, when regulation of position was relaxed (pos → vel → mi), the tendency to increase values of prediction error thresholds and open loop intervals is plausible. For the minimum intervention condition (mi), compliance with the instruction required participants to wait until the position of the unstable system moved close to the edge of the screen before intervening and thus increased thresholds and duration of open loop intervals is expected.

Previous evidence for this task showed the non‐linear remnant to be independent of disturbance amplitude (Levison *et al*. 1969). Our study did not test that finding. While the values of open loop interval are similar for undisturbed pursuit tracking (van de Kamp *et al*. 2013) and the disturbance amplitude used in this experiment, our expectation is that higher amplitudes of disturbance will eventually lead to sampling triggered at the maximum rate physiologically possible (Loram *et al*. 2012). We conclude the key factor determining values of open loop intervals observed here is the order of the system, the goal of the participant, and the intention to control the system output as closely as possible.

### Is predictive open loop control with aperiodic sampling a credible physiological mechanism for manual tracking?

This explanation has the merit that, as a purely deterministic process, it provides an explanation of the total joystick power, linear and non‐linear remnant equal to any alternative explanation and unrivalled by any current, alternative deterministic mechanism. Unlike the linear mechanism, the intermittent control mechanism explained the majority of total power (linear and remnant) (77–87% *vs*. 8–48%, IC *vs* CC). This IC with aperiodic sampling explanation confirms previous findings that did not depend upon any particular model to establish their results: namely (1) that continuous control is neither necessary nor most effective for this task (Loram *et al*. 2011), (2) the numerical values of refractoriness and their increase with system order (van de Kamp *et al*. 2013), (3) the numerical value of minimum open loop intervals in relation to time delays estimated by non‐parametric methods (Loram *et al*. 2009), and (4) the numerical distribution of open loop intervals in relation to distribution of reaction times (cf. Fig. 4, van de Kamp *et al*. 2013).

The idea that the nervous system combines prior information with sensory information to estimate (observe) internal and external states is well established (Bays & Wolpert, 2007; Berniker & Kording, 2011; Proske & Gandevia, 2012). In engineering control, this observer (Fig. 2 *B* and *C*) is an optimal Bayesian state estimator (Kleinman *et al*. 1970). The *hold* requires the estimation of a time varying trajectory of states. The generation of movement related time series has been demonstrated using artificial neural networks trained by unsupervised learning processes (Taylor *et al*. 2007) similar to those present in the human cortex (Doya, 1999, 2000). For discrete, ballistic actions, such as throwing, it is inevitable that time evolving motor signals are pre‐planned and implemented to execute the action. It is not difficult to believe the nervous system performs the function represented by the *hold* process (IC, Fig. 2 *C*).

It is conventional to model sustained sensorimotor control using a linear process with a fixed time delay (van der Kooij & de Vlugt, 2007; Kiemel *et al*. 2011; van der Kooij & Peterka, 2011; Gollee *et al*. 2012). While a fixed transport delay is a reasonable assumption for spinal reflex loops with a temporal jitter of a few milliseconds (Rothwell, 1994), variability in delay increases as one proceeds up the neuraxis through functional stretch reflexes and trans‐cortical responses to voluntary intended reactions (Chan *et al*. 1979; and Fig. 6.5, p. 188 in Brooks, 1986; Pruszynski & Scott, 2012). For manual control of second order systems, the mean feedback loop delay of 180–220 ms places the control process in a substantially different part of the neuraxis compared with, for example, 100 ms for a trans‐cortical response (Pruszynski & Scott, 2012) or 120 ms for visually guided manual control of a 1st order system (Loram *et al*. 2009, 2014). For processes with this mean delay, a distribution of open loop intervals and corresponding visuo‐motor delays is more physiological than a fixed transport delay.

As mentioned above, known mechanisms causing aperiodic threshold related sampling and sampling delays lie in the basal ganglia, parietal cortex and frontal cortex (Cisek & Kalaska, 2005; Gold & Shadlen, 2007; Beck *et al*. 2008; Carpenter *et al*. 2009; Cohen & Frank, 2009; Frank, 2011). These processes are associated with sensorimotor decision making. If we accept that threshold related aperiodic sampling is intrinsic to the process of manual tracking (Navas & Stark, 1968; Miall, 1986; Neilson, 1999; Loram *et al*. 2014), then these neural substrates are implicated as potential locations for that process. The evidence and model based explanation of this experiment constrains this process and their neural substrates to acting at the level of sub‐movements, on a time scale of 200–500 ms, and *within* the sensorimotor loop implementing continuous manual control. For example, one possibility is that cortical and subcortical sensory processing of latency up to 50 ms, provides input to a refractory loop of variable delay (up to 500 ms or more) passing through associated basal ganglia–cerebella loops, prior to initiating each sub‐movement (Houk *et al*. 2007; Frank, 2011; Heenan *et al*. 2014; Loram *et al*. 2014). It requires evidence of intracranial recordings to distinguish this from alternative explanations (e.g. Scott *et al*. 2015) where motor decision making provides external modulatory input to continuous trans‐cortical and subcortical processes, of 50–100 ms latency, which bypass the executive loops through the basal ganglia (Yin & Knowlton, 2006; Loram *et al*. 2014).

### Relevance

Discrete, sequential, reaction time tasks and sensorimotor control tasks are sometimes regarded as different processes with distinct neural substrates (Hardwick *et al*. 2013). We see strength in the view that sustained sensorimotor control proceeds via discrete, sequential processes of threshold related motor decision making. This view sees executive processes of decision making, adaptation of control, and motor selection as occurring at the level of sub‐movements *within* the sensorimotor feedback loop (Ronco *et al*. 1999; Heenan *et al*. 2014; Loram *et al*. 2014, 2016; Loram, 2015) at a temporal scale of 200–500 ms, rather than the more traditional location which is *outside* the inner control loop (Skogestad & Postlethwaite, 1996; Pruszynski & Scott, 2012) at the level of action selection. Extending these executive processes within the feedback loop to the level of sub‐movements gives biological control its characteristic flexibility and adaptability still not observed in engineering systems (Loram *et al*. 2015).

The phenomenon of refractoriness of duration 200 ms or more is currently established to be amodal (e.g. independent of sensory modality – sound, visual, touch; Pashler *et al*. 1998; Dux *et al*. 2006) and is established for a variety of tasks including discrete reaction time tasks (e.g. push button or vocal response to sound, visual stimulus; Pashler *et al*. 1998), speaking (selection of discrete words; Ayora *et al*. 2009), and tasks requiring continuous control including driving (Levy *et al*. 2006; Johns & Cole, 2015), manual pursuit tracking (Craik, 1947), and manual compensatory tracking (Miall *et al*. 1993). As this current paper shows, by comparison with previous work (van de Kamp *et al*. 2013) the values of refractoriness are not altered by the change between pursuit and compensatory tracking or the change resulting from the addition of a multi‐sine disturbance. The limited available evidence suggests the refractory duration of sampling is related to the order of the system controlled, i.e. the complexity of the task (van de Kamp *et al*. 2013) and not to the stability of the system controlled (Loram *et al*. 2011). This evidence suggests to us that our findings are general to voluntary manual control tasks rather than being specific to the particular task, signal and perturbation used in this study.

Investigations into ageing and neurological disorders such as Parkinson's disease show that deterioration in sustained sensorimotor control is strongly associated with deterioration in executive function (Delbaere *et al*. 2010; Schoene *et al*. 2014) and also that Parkinson's disease is associated with abnormal refractoriness (Harrison *et al*. 1995). The methods presented here provide a quantitative means of assessing parameters of motor decision making from sensorimotor tasks such as manual tracking. Hence these methods may be useful in studying the mechanistic basis of the link between executive function and sensorimotor control.

## Appendix: Description of models

### Definition of external and neuro‐muscular system

The human participant controls an external 2nd order or 1st order system using the joystick. The model controls a dynamic system including the external system and a neuromuscular system representing the generation of joystick position from motor output. The external and neuromuscular system are connected in series (Fig. 2).

Figure 2 shows the external 2nd order unstable system, labelled ‘system’ in Fig. 2 *A*–*C*, and a 2nd order linear approximation of the neuro‐muscular system, with a time‐constant of 100 ms, labelled ‘neuromusc. system’. The system is augmented by a disturbance observer with integral action to compensate for any constant disturbances (Gawthrop & Wang, 2009).

### Continuous controller

A standard continuous‐time state‐space controller (Kleinman, 1969; Gawthrop *et al*. 2011) containing an optimal state observer and optimal state feedback, together with a state‐predictor which compensates the delay *t_d_* (representing the mean total delay due to the human operator) is used as a linear continuous‐time predictive controller (CCN) modelling the human operator (Fig. 2 *A* and *B*). Two measured system outputs, *y_o_* (position and velocity), are taken as the observer inputs, together with the control signal u. The state observer and state feedback vectors are designed using standard steady‐state linear quadratic methods, which involve minimising quadratic cost functions of the weighted control signals, system states and output signals (Gollee *et al*. 2012).

### Intermittent controller

The intermittent controller (IC, Fig. 2 *C*) (Gawthrop *et al*. 2011) is based on the same structure as the continuous controller, but instead of continuous feedback of the observer state, feedback is only used at discrete time points, *t*_i_ (indicated by the dashed line). The sampled observer state, *x*_o_(*t_i_*), is fed to a predictor and subjected to a computational delay, *t* ^min^_d = _50 ms (which represents the minimal physiological delay due to the human operator), resulting in the predictor state *x*_p_(*t_i_*). The predictor state is used as the initial condition for a system matched hold element (labelled as ‘hold’) with dynamics which correspond to those of the equivalent continuous time predictive control loop (Gawthrop *et al*. 2011). Thus, in the absence of disturbances, the hold state *x*_h_ follows the observer state *x*_o_. When disturbances or uncertainties affect the loop, *x*_h_ will diverge from *x*_o_, resulting in a non‐zero prediction error, *e*_p_(*t*) = *x*_h_(*t*)−*x*_o_(*t*). A quadratic switching function of the form *e*_p_ *^T^ Q*_t_ *e*_p_ > 1, with *Q*_t_ a positive semi‐definite matrix, can be defined as an event trigger to reset the hold state x_h_ to the observer state *x*_o_ (Gawthrop & Wang, 2009; Gawthrop *et al*. 2014, 2015). In our case only the elements of *e*_p_ corresponding to the velocity (*e*_p_^vel^) and position (*e*_p_^pos^) states are considered, and *Q*_t_ is a diagonal matrix with two positive elements, θ^vel^ (velocity threshold) and θ^pos^ (position threshold), forming the axes of an elliptic switching surface,

(3) ep pos θ pos 2+ep vel θ vel 2>1.

The time between trigger events is the intermittent (or open‐loop) interval, ∆_ol_^i^ = *t_i_*−*t_i_*_−1_. A new trigger event can only occur if ∆_ol_ exceeds a minimal open loop interval, ∆_ol_^min^ > *t_d_* ^min^. In the special case that the thresholds are zero, triggering occurs at a constant interval of ∆_ol_^min^, resulting in regularly sampled IC.

## Additional information

### Competing interests

The authors declare no competing interests.

### Author contributions

H.G., P.J.G., M.L. and I.D.L. conceived and designed the experiments. I.D.L. performed the experiments at Manchester Metropolitan University. H.G., P.J.G. and I.D.L. developed analysis tools and methods. Mathematical and computational modelling was performed by H.G., P.J.G. and I.D.L. The article was drafted by I.D.L. and H.G., and revised critically by P.J.G. and M.L. All authors have approved the final version of the manuscript and all persons designated as authors qualify for authorship, and all those who qualify for authorship are listed.

### Funding

We acknowledge EPSRC financial support for this project via the linked grants EP/F068514/1, EP/F069022/1 and EP/F06974X/1.

## References

[tjp12565-bib-0001] Ayora P , Janssen N , Dell'acqua R & Alario FX (2009). Attentional requirements for the selection of words from different grammatical categories. J Exp Psychol Learn Mem Cogn 35, 1344–1351. 19686027 10.1037/a0016373

[tjp12565-bib-0002] Bays PM & Wolpert DM (2007). Computational principles of sensorimotor control that minimize uncertainty and variability. J Physiol 578, 387–396. 17008369 10.1113/jphysiol.2006.120121 PMC2075158

[tjp12565-bib-0003] Beck JM , Ma WJ , Kiani R , Hanks T , Churchland AK , Roitman J , Shadlen MN , Latham PE & Pouget A (2008). Probabilistic population codes for Bayesian decision making. Neuron 60, 1142–1152. 19109917 10.1016/j.neuron.2008.09.021 PMC2742921

[tjp12565-bib-0004] Beggs JM & Plenz D (2003). Neuronal avalanches in neocortical circuits. J Neurosci 23, 11167–11177. 14657176 10.1523/JNEUROSCI.23-35-11167.2003 PMC6741045

[tjp12565-bib-0005] Berniker M & Kording K (2011). Bayesian approaches to sensory integration for motor control. Wiley Interdisciplinary Reviews: Cognitive Science 2, 419–428. 26302201 10.1002/wcs.125

[tjp12565-bib-0006] Brooks VB (1986). The Neural Basis of Motor Control. Oxford University Press, Oxford.

[tjp12565-bib-0007] Caligiore D , Pezzulo G , Baldassarre G , Bostan AC , Strick PL , Doya K , Helmich RC , Dirkx M , Houk J , Jorntell H , Lago‐Rodriguez A , Galea JM , Miall RC , Popa T , Kishore A , Verschure PF , Zucca R & Herreros I (2017). Consensus paper: towards a systems‐level view of cerebellar function: the interplay between cerebellum, basal ganglia, and cortex. Cerebellum 16, 203–229. 26873754 10.1007/s12311-016-0763-3 PMC5243918

[tjp12565-bib-0008] Carpenter RHS , Reddi BAJ & Anderson AJ (2009). A simple two‐stage model predicts response time distributions. J Physiol 587, 4051–4062. 19564395 10.1113/jphysiol.2009.173955 PMC2756437

[tjp12565-bib-0009] Chan CW , Kearney RE & Jones GM (1979). Tibialis anterior response to sudden ankle displacements in normal and Parkinsonian subjects. Brain Res 173, 303–314. 487092 10.1016/0006-8993(79)90630-9

[tjp12565-bib-0010] Cisek P & Kalaska JF (2005). Neural correlates of reaching decisions in dorsal premotor cortex: Specification of multiple direction choices and final selection of action. Neuron 45, 801–814. 15748854 10.1016/j.neuron.2005.01.027

[tjp12565-bib-0011] Cohen MX & Frank MJ (2009). Neurocomputational models of basal ganglia function in learning, memory and choice. Behav Brain Res 199, 141–156. 18950662 10.1016/j.bbr.2008.09.029 PMC2762323

[tjp12565-bib-0012] Craik KJW (1947). Theory of the human operator in control systems. I. The operator as an engineering system. Br J Psychol xxxviii, 56. 10.1111/j.2044-8295.1947.tb01141.x 18917476

[tjp12565-bib-0013] Delbaere K , Close JC , Heim J , Sachdev PS , Brodaty H , Slavin MJ , Kochan NA & Lord SR (2010). A multifactorial approach to understanding fall risk in older people. J Am Geriatr Soc 58, 1679–1685. 20863327 10.1111/j.1532-5415.2010.03017.x

[tjp12565-bib-0014] Dideriksen JL , Negro F , Enoka RM & Farina D (2012). Motor unit recruitment strategies and muscle properties determine the influence of synaptic noise on force steadiness. J Neurophysiol 107, 3357–3369. 22423000 10.1152/jn.00938.2011 PMC3378401

[tjp12565-bib-0015] Doya (1999). What are the computations of the cerebellum, the basal ganglia and the cerebral cortex? Neural Netw 12, 961. 12662639 10.1016/s0893-6080(99)00046-5

[tjp12565-bib-0016] Doya K (2000). Complementary roles of basal ganglia and cerebellum in learning and motor control. Curr Opin Neurobiol 10, 732. 11240282 10.1016/s0959-4388(00)00153-7

[tjp12565-bib-0017] Dux PE , Ivanoff J , Asplund CL & Marois R (2006). Isolation of a central bottleneck of information processing with time‐resolved fMRI. Neuron 52, 1109–1120. 17178412 10.1016/j.neuron.2006.11.009 PMC2527865

[tjp12565-bib-0018] Frank MJ (2011). Computational models of motivated action selection in corticostriatal circuits. Curr Opin Neurobiol 21, 381–386. 21498067 10.1016/j.conb.2011.02.013

[tjp12565-bib-0019] Fuglevand AJ , Winter DA & Patla AE (1993). Models of recruitment and rate coding organization in motor‐unit pools. J Neurophysiol 70, 2470–2488. 8120594 10.1152/jn.1993.70.6.2470

[tjp12565-bib-0020] Gawthrop P , Gollee H & Loram I (2015). Intermittent control in man and machine In Event‐based Control and Signal Processing, http://arxiv.org/abs/1407.3543. CRC Press/Taylor & Francis.

[tjp12565-bib-0021] Gawthrop P , Loram I , Gollee H & Lakie M (2014). Intermittent control models of human standing: similarities and differences. Biol Cybern 108, 159–168. 24500616 10.1007/s00422-014-0587-5 PMC3962584

[tjp12565-bib-0022] Gawthrop P , Loram I & Lakie M (2009). Predictive feedback in human simulated pendulum balancing. Biol Cybern 101, 131–146. 19588160 10.1007/s00422-009-0325-6

[tjp12565-bib-0023] Gawthrop P , Loram I , Lakie M & Gollee H (2011). Intermittent control: a computational theory of human control. Biol Cybern 104, 31–51. 21327829 10.1007/s00422-010-0416-4

[tjp12565-bib-0024] Gawthrop P & Wang L (2011). The system‐matched hold and the intermittent control separation principle. Int J Control 84, 1965–1974.

[tjp12565-bib-0025] Gawthrop PJ & Wang L (2009). Event‐driven intermittent control. Int J Control 82, 2235–2248.

[tjp12565-bib-0026] George GR (2008). New Methods of Mathematical Modeling of Human Behavior in the Manual Tracking Task, pp. 340 State University of New York at Binghamton, Ann Arbor.

[tjp12565-bib-0027] Gold JI & Shadlen MN (2007). The neural basis of decision making. Annu Rev Neurosci 30, 535–574. 17600525 10.1146/annurev.neuro.29.051605.113038

[tjp12565-bib-0028] Gollee H , Mamma A , Loram I & Gawthrop P (2012). Frequency‐domain identification of the human controller. Biol Cybern 106, 359–372. 22798036 10.1007/s00422-012-0503-9

[tjp12565-bib-0029] Haber SN , Fudge JL & McFarland NR (2000). Striatonigrostriatal pathways in primates form an ascending spiral from the shell to the dorsolateral striatum. J Neurosci 20, 2369–2382. 10704511 10.1523/JNEUROSCI.20-06-02369.2000 PMC6772499

[tjp12565-bib-0078] Halliday DM , Rosenberg JR , Amjad AM , Breeze P , Conway BA & Farmer SF (1995). A framework for the analysis of mixed time series/point process data–theory and application to the study of physiological tremor, single motor unit discharges and electromyograms. Prog Biophys Mol Biol 64, 237–278. 8987386 10.1016/s0079-6107(96)00009-0

[tjp12565-bib-0030] Hamilton AF , Jones KE & Wolpert DM (2004). The scaling of motor noise with muscle strength and motor unit number in humans. Exp Brain Res 157, 417–430. 15014922 10.1007/s00221-004-1856-7

[tjp12565-bib-0031] Hardwick RM , Rottschy C , Miall RC & Eickhoff SB (2013). A quantitative meta‐analysis and review of motor learning in the human brain. NeuroImage 67, 283–297. 23194819 10.1016/j.neuroimage.2012.11.020 PMC3555187

[tjp12565-bib-0032] Harris CM & Wolpert DM (1998). Signal‐dependent noise determines motor planning. Nature 394, 780. 9723616 10.1038/29528

[tjp12565-bib-0033] Harrison J , Henderson L & Kennard C (1995). Abnormal refractoriness in patients with Parkinson's disease after brief withdrawal of levodopa treatment. J Neurol Neurosurg Psychiatry 59, 499–506. 8530934 10.1136/jnnp.59.5.499 PMC1073712

[tjp12565-bib-0034] Heenan M , Scheidt RA , Woo D & Beardsley SA (2014). Intention tremor and deficits of sensory feedback control in multiple sclerosis: a pilot study. J Neuroeng Rehabil 11, 170. 25526770 10.1186/1743-0003-11-170 PMC4292988

[tjp12565-bib-0035] Houk JC , Bastianen C , Fansler D , Fishbach A , Fraser D , Reber PJ , Roy SA & Simo LS (2007). Action selection and refinement in subcortical loops through basal ganglia and cerebellum. Philos Trans R Soc Lond B Biol Sci 362, 1573–1583. 17428771 10.1098/rstb.2007.2063 PMC2440782

[tjp12565-bib-0036] Insperger T & Milton J (2014). Sensory uncertainty and stick balancing at the fingertip. Biol Cybern 108, 85–101. 24463637 10.1007/s00422-013-0582-2

[tjp12565-bib-0037] Jagacinski R & Flach J (2003). Control Theory for Humans: Quantitative Approaches to Modeling Performance. Lawrence Erlbaum Associates, Publishers, Mahwah, NJ, USA.

[tjp12565-bib-0038] Johns TA & Cole DJ (2015). Measurement and mathematical model of a driver's intermittent compensatory steering control. Veh Syst Dyn 53, 1811–1829.

[tjp12565-bib-0039] Jones KE , Hamilton AF & Wolpert DM (2002). Sources of signal‐dependent noise during isometric force production. J Neurophysiol 88, 1533–1544. 12205173 10.1152/jn.2002.88.3.1533

[tjp12565-bib-0040] Kiemel T , Zhang Y & Jeka JJ (2011). Identification of neural feedback for upright stance in humans: stabilization rather than sway minimization. J Neurosci 31, 15144–15153. 22016548 10.1523/JNEUROSCI.1013-11.2011 PMC3470452

[tjp12565-bib-0041] Kleinman DL (1969). Optimal control of linear systems with time‐delay and observation noise. IEEE Transactions on Automatic Control 14, 524–527.

[tjp12565-bib-0042] Kleinman DL , Baron S & Levison WH (1970). An optimal control model of human response 1. Theory and validation. Automatica 6, 357–369.

[tjp12565-bib-0043] Levison WH , Baron S & Kleinman DL (1969). A model for human controller remnant. IEEE Transactions on Man‐Machine Systems 10, 101–108.

[tjp12565-bib-0044] Levy J , Pashler H & Boer E (2006). Central interference in driving: is there any stopping the psychological refractory period? Psychol Sci 17, 228–235. 16507063 10.1111/j.1467-9280.2006.01690.x

[tjp12565-bib-0045] Loram I (2015). Postural control and sensorimotor integration In Grieve's Modern Musculoskeletal Physiotherapy, 4th edn, eds Jull GA, Moore A, Falla D, Lewis J, McCarthy C & Sterling M Elsevier.

[tjp12565-bib-0046] Loram I , Bate B , Harding P , Cunningham R & Loram A (2016). Proactive selective inhibition targeted at the neck muscles: this proximal constraint facilitates learning and regulates global control. IEEE Trans Neural Syst Rehab Eng 25, 357–369. 10.1109/TNSRE.2016.2641024 28026778

[tjp12565-bib-0047] Loram I , Gawthrop P & Gollee H (2015). Intermittent control of unstable multivariate systems. In *Engineering in Medicine and Biology Society (EMBC), 2015 37th Annual International Conference of the IEEE*, pp. 1436–1439. 10.1109/EMBC.2015.7318639 26736539

[tjp12565-bib-0048] Loram ID , Gollee H , Lakie M & Gawthrop PJ (2011). Human control of an inverted pendulum: is continuous control necessary? Is intermittent control effective? Is intermittent control physiological? J Physiol 589, 307–324. 21098004 10.1113/jphysiol.2010.194712 PMC3043535

[tjp12565-bib-0049] Loram ID , Lakie M & Gawthrop PJ (2009). Visual control of stable and unstable loads: what is the feedback delay and extent of linear time‐invariant control? J Physiol 587, 1343–1365. 19171654 10.1113/jphysiol.2008.166173 PMC2675002

[tjp12565-bib-0050] Loram ID , van de Kamp C , Gollee H & Gawthrop PJ (2012). Identification of intermittent control in man and machine. J R Soc Interface 9, 2070–2084. 22491973 10.1098/rsif.2012.0142 PMC3405763

[tjp12565-bib-0051] Loram ID , van de Kamp C , Lakie M , Gollee H & Gawthrop PJ (2014). Does the motor system need intermittent control? Exerc Sport Sci Rev 42, 117–125. 24819544 10.1249/JES.0000000000000018

[tjp12565-bib-0052] McLachlan G & Peel D (2000). Finite Mixture Models. John Wiley & Sons, Inc, Hoboken, NJ, USA.

[tjp12565-bib-0053] Miall RC (1986). Simple or complex‐systems. Behav Brain Sci 9, 734–734.

[tjp12565-bib-0054] Miall RC , Weir DJ & Stein JF (1986). Manual tracking of visual targets by trained monkeys. Behav Brain Res 20, 185–201. 3730133 10.1016/0166-4328(86)90003-3

[tjp12565-bib-0055] Miall RC , Weir DJ & Stein JF (1993). Intermittency in human manual tracking tasks. J Mot Behav 25, 53. 12730041 10.1080/00222895.1993.9941639

[tjp12565-bib-0056] Navas F & Stark L (1968). Sampling or intermittency in hand control system dynamics. J Biophysics 8, 252–302. 10.1016/S0006-3495(68)86488-4 PMC1367375 5639937

[tjp12565-bib-0057] Neilson PD (1999). Influence of intermittency and synergy on grasping. Motor Control 3, 280. 10409800 10.1123/mcj.3.3.280

[tjp12565-bib-0058] Newell KM , Deutsch K , Sosnoff J & Mayer‐Kress G (2006). Variability in motor output as noise: a default and erroneous proposition? In Movement System Variability, eds Davids K, Bennett S & Newell KM Human Kinetics, USA.

[tjp12565-bib-0059] Pashler H , Johnston JC & Pashler H (1998). Attentional limitations in dual‐task performance In Attention, pp. 155 Psychology Press, Hove.

[tjp12565-bib-0060] Pataky TC (2012). One‐dimensional statistical parametric mapping in Python. Comput Methods Biomech Biomed Engin 15, 295–301. 10.1080/10255842.2010.527837 21756121

[tjp12565-bib-0061] Pataky TC , Robinson MA & Vanrenterghem J (2013). Vector field statistical analysis of kinematic and force trajectories. J Biomech 46, 2394–2401. 23948374 10.1016/j.jbiomech.2013.07.031

[tjp12565-bib-0062] Patzelt F , Riegel M , Ernst U & Pawelzik K (2007). Self‐organized critical noise amplification in human closed loop control. Front Comput Neurosci 1, 4. 18946526 10.3389/neuro.10.004.2007 PMC2525932

[tjp12565-bib-0063] Pintelon R & Schoukens J (2001). System Identification: A Frequency Domain Approach. Institute of Electrical and Electronic Engineers, New York.

[tjp12565-bib-0064] Prochazka A (2000). Muscle afferent activity and reflex actions during cat locomotion. J Physiol 525, 6S.

[tjp12565-bib-0065] Proske U & Gandevia SC (2012). The proprioceptive senses: their roles in signaling body shape, body position and movement, and muscle force. Physiol Rev 92, 1651–1697. 23073629 10.1152/physrev.00048.2011

[tjp12565-bib-0066] Pruszynski JA & Scott SH (2012). Optimal feedback control and the long‐latency stretch response. Exp Brain Res 218, 341–359. 22370742 10.1007/s00221-012-3041-8

[tjp12565-bib-0067] Rochester L , Lord S , Yarnall AJ & Burn DJ (2014). Falls in patients with dementia In Movement Disorders in Dementias, eds Merello M & Starkstein ES, pp. 45–60. Springer London, London.

[tjp12565-bib-0068] Ronco E , Arsan T & Gawthrop PJ (1999). Open‐loop intermittent feedback control: practical continuous‐time GPC. IEE Proceedings ‐ Control Theory and Applications 146, 426–434.

[tjp12565-bib-0069] Rothwell J (1994). Control of Human Voluntary Movement. Chapman & Hall, London.

[tjp12565-bib-0070] Schoene D , Valenzuela T , Lord SR & de Bruin ED (2014). The effect of interactive cognitive‐motor training in reducing fall risk in older people: a systematic review. BMC Geriatr 14, 107. 25240384 10.1186/1471-2318-14-107 PMC4181419

[tjp12565-bib-0071] Scott SH , Cluff T , Lowrey CR & Takei T (2015). Feedback control during voluntary motor actions. Curr Opin Neurobiol 33, 85–94. 25827274 10.1016/j.conb.2015.03.006

[tjp12565-bib-0072] Skogestad S & Postlethwaite I (1996). Multivariable Feedback Control Analysis and Design. John Wiley & Sons, Inc, New York.

[tjp12565-bib-0073] Taylor G , Hinton GE & Rowels S (2007). Modelling human motion using binary latent variables In Advances in Neural Information Processing Systems, eds Schölkopf PB, Platt JC & Hoffman T, Proceedings from Neural Information Processing Systems 2006. Neural Information Processing Systems Foundation, Inc.

[tjp12565-bib-0074] van de Kamp C , Gawthrop PJ , Gollee H & Loram ID (2013). Refractoriness in sustained visuo‐manual control: is the refractory duration intrinsic or does it depend on external system properties? PLoS Comput Biol 9, e1002843. 23300430 10.1371/journal.pcbi.1002843 PMC3536613

[tjp12565-bib-0075] van der Kooij H & de Vlugt E (2007). Postural responses evoked by platform pertubations are dominated by continuous feedback. J Neurophysiol 98, 730–743. 17460106 10.1152/jn.00457.2006

[tjp12565-bib-0076] van der Kooij H & Peterka RJ (2011). Non‐linear stimulus‐response behavior of the human stance control system is predicted by optimization of a system with sensory and motor noise. J Comput Neurosci 30, 759–778. 21161357 10.1007/s10827-010-0291-y PMC3108015

[tjp12565-bib-0077] Yin HH & Knowlton BJ (2006). The role of the basal ganglia in habit formation. Nat Rev Neurosci 7, 464–476. 16715055 10.1038/nrn1919

